# Playing Online as Preparation for Mathematics: The Cultural-Historical Approach as an Alternative to Constructivism

**DOI:** 10.11621/pir.2023.0305

**Published:** 2023-09-30

**Authors:** Yulia Solovieva, Luis Quintanar, Valeriya A. Plotnikova

**Affiliations:** a Autonomous University of Puebla, Mexico; b Autonomous University of Tlaxcala, Mexico; c Lomonosov Moscow State University, Moscow, Russia

**Keywords:** introduction of mathematics, symbolic function, online methodology, innovative developmental methods, preschool development

## Abstract

**Background:**

While the traditional method of teaching consists of repetition and memorization, the constructivist theory proposes independent discovery and free play. The cultural-historical approach, on the other hand, does not insist on the early introduction of formal mathematics as implicit or explicit knowledge. According to this outlook, important psychological developmental is necessary for the child before he/she can learn mathematics in primary school.

**Objective:**

To present a methodology for organizing the play activity of children of preschool age by introducing symbolic means on the materialized and perceptual levels as an essential aspect of preparation for learning mathematical concepts in primary school.

**Design:**

The experiment consisted in pedagogical work in the online modality by the authors at a private college in the city of Puebla, Mexico. Eighteen children from three levels of preschool education (from three to six years old) participated in 45-minute sessions three times per week. There were six children from each preschool level.

**Results:**

It was shown that playing with roles online allows children at least partially to include different symbolic means in their activity. This allows the children not only to satisfy their curiosity and be positively engaged in the topic of the play, but also to develop symbolic functions as preparation for intellectual actions with numerical content in primary school.

**Conclusion:**

The article shows a feasible way to organize preschool play with roles online and thus to scaffold the formation of children’s imagination and ability to use symbolic means, which is important for future learning. The cultural-historical approach offers useful guidelines here, although more research is needed to support the development of children’s symbolic function within math-specific activity, based on activity theory applied to learning.

## Introduction

The question of how to begin teaching mathematics to youth is a matter of concern for psychologists, educators, and teachers internationally. In Latin America, assessments of students mathematical knowledge by international organizations persist in yielding very poor results (OGDE, 2016). This is caused by a profound crisis in the system of education, and the inability of educators to provide successful work with mathematical knowledge at the preschool age and in primary school. The predominant tendency of both public and private preschool and primary school institutions is to adopt the constructivist point of view of learning (SEP, 2017). In Brazil, the largest Latin American country, the most recent program of the Ministry of Education claimed that no special teaching of mathematics is necessary, because all children learn through interaction and communication in different contexts (Ministry of Education of Brazil, 2021). In the countries of Latin America, the constructivist approach to the teaching and learning process is frequently confused with the cultural-historical approach (Solovieva et al., 2022).

According to the constructivist approach, each child’s learning is an independent and spontaneous process. Children construct their knowledge through broad social interaction in the family, the street, and the park, as well as in the classroom. School is considered as just one social institution among many others (Calva, Quijano, & Estrella, 2018). The role of the teacher is to facilitate and create conditions for development, but not to be responsible for this development. The teacher is a kind of observer, one participant among many others in the educational process, with no specific developmental role.

The constructivist approach also asserts that the primary requirement for development is to provide sensory enrichment for different perceptual modalities through contact with various objects, substances, and textures, so that the preschool classroom should have the relevant objects. Such objects and substances should have the physical characteristics of extension (length), dimension (area and volume), weight, and the space that they occupy; the objects might be counted and gathered into groups or combinations, and these combinations might be organized in different ways. At first glance, the constructivist position seems to be child-friendly and appropriate for early learning and for positive social interaction at preschool age. Each child may take any attractive object or toy, put it somewhere, exchange it with another child, or give it to the teacher and receive positive encouragement. There are no grades or long homework tasks, at least at preschool age. The whole atmosphere is positive and inclusive for all children. This method is broadly used not only in Mexico, but also in many other countries, in both private and public institutions; it is considered the best example of an active and innovative method for preschool and early school for developing mathematical abilities (Navarra & Larrea, 2018).

However, there are some weak points in the constructivist position. The first is that teaching and learning are understood as two isolated, separate processes. The role of the adult is only to assist and provide the sensory conditions for exploration (Luria, 2019). The second weakness is the lack of differentiation between non-specific exploration and manipulation of an object with no specific goal, and actions with cultural objectives. The third weakness is that there is no distinction between empirical and theoretical-scientific concepts and the method of their introduction in preschool age and in primary school (Kazanskaya & Romashchuk, 2021). The children’s work with concepts is always empirical, which means that they never grasp the mathematical concepts behind what they are learning. Teachers are convinced that empirical knowledge will lead spontaneously to theoretical knowledge. It is not at all clear how the child may arrive independently at some mathematical actions and concepts just by manipulating objects and textures An educator thus provides necessary conditions for learning, but never actually conveys knowledge (Montessori, 2004).

All actions in constructivist methodology are called games, but it is not clear what the word “game” means, since there are no explicit rules, motives, or goals for the collective realization of exploration and manipulation. Any kind of manipulative operation by the child with any object is called a “game.” Specific mathematical content, such as quantity, measuring, the physical characteristics of objects, correspondence, seriation, and equalization, are implicitly included in the concrete material, supposedly providing the possibility of the child’s spontaneous discovery of them. But there is no specific guidance for work with these materials.

Another serious problem is the absence of a clear relationship between numerical concepts and the representation of reality that stands behind these concepts. Each child can manipulate and explore each object while having no clarity about basic mathematical concepts. At the same time, it is not clear that this kind of exploration furthers the children’s psychological development. The children experience joyful and friendly lessons, but never learn what the goal of mathematics is, what the measuring expresses, and why different signs and symbols are used.

In Mexico, as in other Latin American countries during the international pandemic, a real crisis began at all levels of the educational system, but especially at the preschool level. The constructivist approach showed its complete inability to provide adequate lessons online. All children were isolated and stayed at home for more than one year. Interaction with concrete sensory materials for spontaneous use by the children was not accessible in their day-to-day lives. Some children were able to use technology and take online classes, but most of them had extreme difficulties. Even children who had access to electronic technologies could not participate in long class periods online. Most young children in Mexico did not have such access, especially those attending public schools.

The major obstacle to teaching preschool children online was that the teachers had no specific methodology for working online (and still do not today). Recent studies have shown that preschool children have difficulty working with tactile recognition of objects after long periods of work with virtual media (Tikhomirova, Malykh, & Malykh, 2020). The only methodology used by teachers in Mexico was to send assignments to the children by WhatsApp, phone, or video calls, so that the parents became very busy receiving and fulfilling “home tasks” for long hours. Very few educational institutions were able to give continuous classes online.

The method of teaching online proposed in this article consists in organizing simultaneous sessions of play activity for preschoolers and general intellectual activities in the first grade of primary school as a collectively shared and guided activity. Such activities permit the introduction and strengthening at preschool age of psychological preparation for school and the introduction of basic mathematical knowledge, which is the foundation for successful future learning of mathematics in primary school. Such work can only be provided in simultaneous online sessions by mutual work between the teacher and the children and might be accomplished with very little homework; that homework is always to be explained and checked by the teacher (Olivera Montaño et al, 2021; Solovieva & Quintanar, 2021). The whole conception of the use of play activity at preschool age is related to the meaningful periods of psychological development introduced by Vygotsky (1996) and continued by his followers (Elkonin, 1995; Fleer, 2022). Specifically, the periods of psychological development are understood as qualitative and long periods, each of which has its own guiding activity. Preschool age is a period of psychological development directed to formation of voluntary and symbolic activity (Elkonin, 1995; Salmina, 2017; Talyzina, 2019). It is possible to say that playing with roles is not specific preparation for mathematics, but general psychological preparation for intellectual activity, which is also useful for mathematics.

The goal of this article is to show the method for organizing play activity online for children of preschool age, which will prepare them for the introduction of general mathematical concepts based on the cultural-historical approach and psychological activity theory. The method, based on Elkonin’s conception of playing activity (Elkonin, 1995), was applied during the pandemic in groups of preschool children in Mexico. The examples are provided from the authors’ practical work under the direction of Kepler College in the city of Puebla. The authors are organizers and pedagogical advisers at the college and collected and organized the data obtained during the 2020–2021 pandemic.

## Preparation for Mathematics Through Online Play Activity

The Mexican Education System mandates preschool education starting at the age of three, and continuing for three years. All children enter primary school at the age of six. The program for preschool education includes introduction of mathematical knowledge in an implicit way, according to the constructivist approach (SEP, 2017).

Another proposal for preschool development comes from the cultural-historical theory introduced to psychology by L.S. Vygotsky. According to research conducted following this approach, the key components of psychological preparation for school are voluntary activity, imagination, self-reflection, and symbolic function (Salmina, 2013a, 2013b; Veraksa et al., 2022; Yudina, 2022). Symbolic function might be considered the basis for the proper introduction of mathematics at school (Davydov, 2000; Salmina, 2017). The problem in developing this function is that it is impossible to teach it directly as one would teach formal school subjects. Symbolic means (signs and symbols) can be used only for specific actions, which should not only be practical actions with material objects. Actions that require the use of symbolic means are actions of play activity and elementary intellectual actions. The play actions represent the content of the play activity, which promote the psychological development of the children, while at primary school age, intellectual activity becomes the central line of psychological development (Davydov, 2000).

During preschool, symbolic function might be broadly used in different kinds of play activity, such as table games, active games, and playing with roles. This last type of play activity is especially interesting from the psychological point of view, because it represents an excellent example of collective and communicative activity, which is so useful for preschool development. Putting on a play involves the use of verbal and non-verbal actions to represent chosen roles in imaginary social situations according to a chosen topic or argument (Solovieva & Quintanar, 2019). It is possible to include a broad diversity of symbolic means in these imaginary situations. These might be of different levels of complexity, ranging from substitution to codification (regulation) of behavior, schematization, and modeling (Salmina, 1988, 2013b). Each new level subsumes the previous one: for example, codification is based on substitution; schematization includes substitution and codification; modeling uses substitution, codification, and schematization (*Table 1*). The levels might also be understood as different types of symbolic operations, since they might be used in different activities and situations on different subjects.

**Table 1:** Types of symbolic operations in role playing

Type of symbolic operation	Content of the play	Example of action in the play
Substitution	One object is used instead of another	A small ball as a microphone
Codification / Regulation	Specific signs, which represent the rules or norms of action or behavior	Symbols, which show whether the Zoo is open or closed
Schematization	Plans, maps, and signs, which represent directions or distribution of elements in the space	Directions to follow in the Zoo
Modeling	Objects, symbolic means for rules and for movement and distribution in space, united in a global representation of the whole situation (complex objective)	The model of the Zoo

The children themselves are not aware of the types or levels of symbolic functions, but they understand in general that they are representing and using different symbols in the play. Such a semi-conscious level of functioning might be called operations, according to the activity theory model, where the actions are psychological processes which are directed to conscious goals according to a predominant general motive (Leontiev, 1975). In this psychological model, the differentiation between operations and actions is one of the central aspects of cultural activity. The action is directed to a reflective, conscious goal, while the operation implies semi-conscious use of the material and physical means (including objects, gestures, and movements) which are necessary to achieve the goal of the action within the play or intellectual activity.

Such differentiation is useful for understanding the process of symbolic development in childhood, and for establishing its relationship with intellectual development and preparation for learning at school. At the stage of substitution, we propose to refer to representative use of symbols in play as symbolic operations instead of symbolic actions, because the children do not follow the conscious goal of choosing specific symbolic means, but of fulfilling external representative action with an objective in mind.

The symbols are the means which serve to achieve another goal: communicative expression of the content of the role in a way understandable to another participant in the play. *Table 1* presents the types of symbolic operations which might be used in plays with roles.

While following the stages of development in putting on a play, such as codification, schematization, and modeling, the children start to perform symbolic actions, in which the goal is to create or choose the proper symbolic means before they can realize the actions of representation. In this case, the whole symbolic function may include different actions of election, creation, and valuation of symbolic means before the action of representation of a role in the play. On the other hand, at the beginning, at the stage of simple substitution, there is no creation of symbolic means; at this early stage, the children may only use external objects as substitutes for other objects. In the later stages, the children transform the operation of substitution into an act of creation of symbolic means.

Our previous studies have confirmed that symbolic means might be introduced by a teacher or created by the children during the stage of orientation and discussion before starting the process of putting on the play (Solovieva & Gonzales, 2017; Veraksa et al., 2022). Symbolic means might be introduced on the materialized or perceptual levels (Solovieva, Gonzales, & Quintanar, 2015). The possibility of introducing or creating symbolic means depends on the age of the children and on their experience with play activity in groups; constant participation in dialogues and active representation using objects and means allow children to achieve a high level of symbolic development. It is obvious that such a process of using symbolic means might be also called “actions.” The children use symbolic means during play actions.

**Figure 1.**
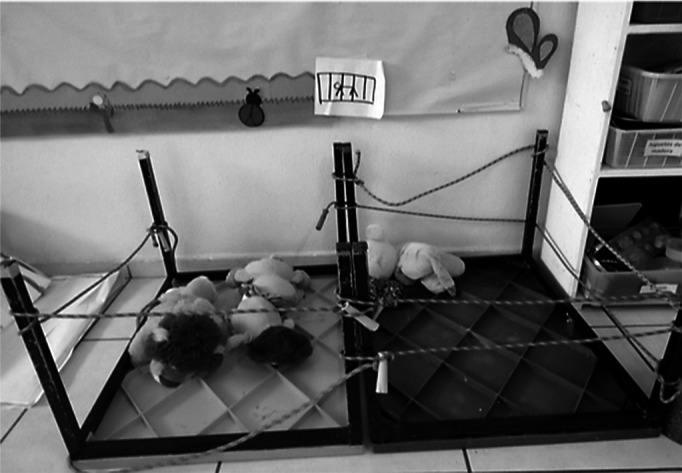
The play about *The Zoo*

*Figure 1* shows a face-to-face session of children putting on a play with roles on the topic of “The Zoo’’ in an experimental kindergarten before the pandemic (www.colegiokepler.edu.mx). The use of symbolic means might be appreciated in this example, as the chairs represent “the cages” in the Zoo and the picture shows the cage with “wild big mammals”.

## Method: Playing with Roles Online

The social and educational isolation experienced during the pandemic was a serious obstacle not only for communication in groups, but also for symbolic development by preschool children. The possibility of organizing simultaneous online classes for groups of preschool children was the only option for continuing playing with roles during the pandemic. The sessions of play activity with roles have their own proper organization (Solovieva & Quintanar, 2019). How was it possible to transfer such a methodology into online sessions, conserving the importance of the use and creation of symbols and signs?

### Participants

The method of online play sessions included orientation for playing activity and the process of play on each topic during collective dialogue and playing actions of children with the toys and other objects during the 45-minute sessions. Children from all three levels of preschool education (from ages three to six) were included in these sessions three times per week during the pandemic, in separate groups. There were six children per group from each preschool level, for a total of 18 children. Each teacher worked with groups of children from the first, second, and third level of preschool education.

### Procedure

All children took part in the simultaneous online sessions; on some occasions, the parents also participated, if their occupations allowed. All sessions were divided between orientation and realization of the play’s activity.

The topic of the play, the roles, the actions, the objects, and possible symbols and signs were discussed during orientation. Each child, in front of the computer at home, selected the toys and the objects to use for the next session. All the elements and the procedure of the play were analyzed collectively by all the children. The sessions dedicated to playing activity took place with all participants, teacher, and children, each child at home with his or her own toys, objects, and external symbols. The children used material objects and toys at home and represented actions according to the role chosen for thematic play during the orientation and discussion. The children demonstrated all the actions of the play and representations on screen to the other participants and directed their oral expressions to the other “roles’’ (participants) in the play.

The whole situation was organized as if it were a face-to-face performance of a play. The children were introduced by a teacher to the social situation (for example, a pastry shop), the roles of this situation or topic (cook, clients, seller, receptionist, and so on), and the actions of each role (to cook, to buy, to offer, to pay, and so on). The teacher and the children decided about the objects, toys, and symbols, necessary for each topic. After explanation, the roles were distributed among the children, who then performed the roles in front of the computer, so that each participant could observe the actions and hear the oral expressions of the others.

Obviously, it is impossible to equate this activity with face-to-face sessions, because of the absence of direct eye, corporal, postural, and kinetic contact among all participants. It is very important to stress that such contact is essential for the psychological development of preschool age children.

## Results: The Effects of Online Inclusion of Symbolic Means, Essential Preparation for Learning Mathematics

Playing with roles online allows children at least partially to include different symbolic means in the content, although it is not possible to say that these procedures are the same as it in a face-to-face modality. The children need to share a real material space to exchange their ideas about creating symbolic media during online sessions. At the same time, in online sessions, the primary necessity is to explore and use specific material actions with objects and toys, so that all the children want to show and talk about their toys and the roles they have chosen.

In addition, online sessions of play activity with roles allowed the exchange of emotional expressions among the participants, by contrast with their loneliness at home during the pandemic. The children often got excited while playing their roles, and expressed positive emotions and positive relationships with each other and the adults during this online play situation.

**Figure 2.**
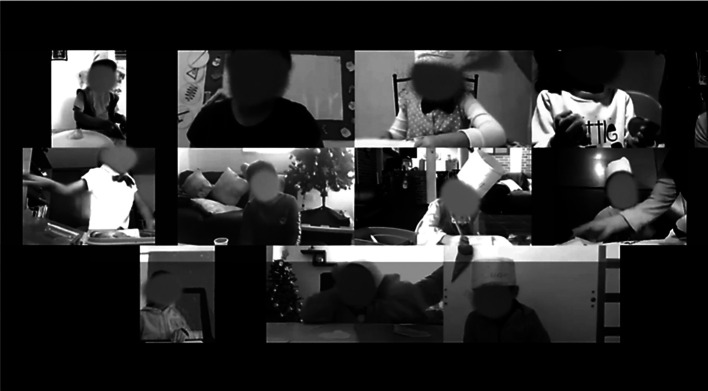
The play *Pastry Shop*

*Figure 2* shows how the children showed their toys and objects to the other participants on screen, according to the chosen topic for the play. They used clothing as the attributes of their roles in the play. The figure shows the possibility of organizing children’s joint role-playing online. It is important to stress that play activity online requires not only the constant inclusion of collective dialogue between the children and the educator on the topic of the play, but also the use of material objects and symbolic means. “Pure” online play makes no sense to children of preschool age.

The teacher noticed that during online sessions, the children performed the roles with great pleasure and interest.

**Figure 3.**
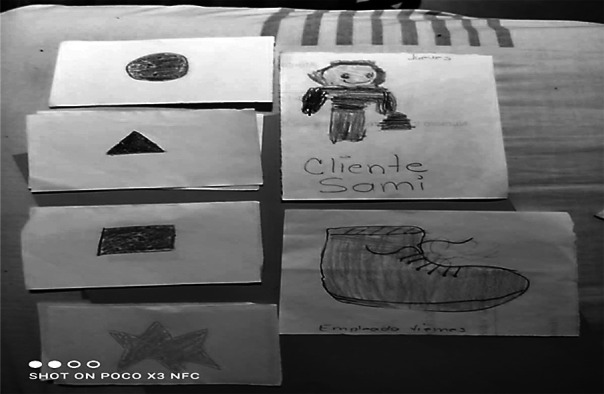
The symbols in the play *Shoe Store.*

In this unusual situation of online playing, the symbolic function takes second place to the content of the play, and requires more time and effort from the teacher. The teacher must put more emphasis on the implementation and collective creation of different symbolic means in the online modality than if the children were physically together. While putting on a play with roles in a face-to-face modality offers the broad possibility for the use and creation of symbolic means during the operations of substitution, codification, regulation, schematization, and modeling, it is necessary to modify this process for the online modality. *Figure 3* shows the use of symbolic means in a play with roles in the online modality, created by the children and used during the play.

**Figure 4.**
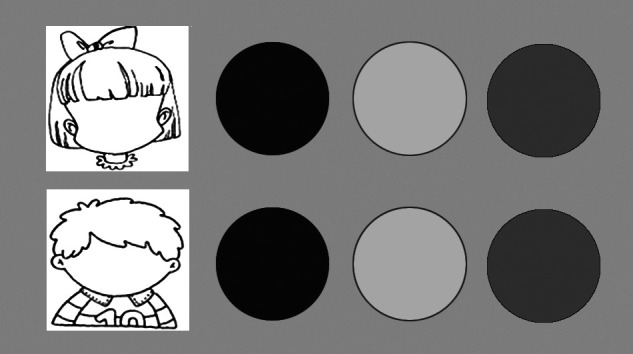
Symbols in the play *Hospital*

In this example, you see the symbols for the play “Shoe Store” being used by the children. Each child prepared the symbols at home and showed the results to everybody on the screen. Examples of the children’s actions during the play were photographed by the parents and sent to the teacher by e-mail (*Figure 3*). This example shows the use of symbols for the operation of codification: the “client” of the “store” may look for different types of shoes, represented with different colors and geometric figures. The symbolic operation of codification refers to representing the content of the play with the help of symbols that stand for different store departments. *Figure 4* shows another use of symbolic means in a play.

In *Figure 4*, we see how another operation of codification is used to determine the illness of a patient in a clinic. The red color designates patients with severe disease, yellow is for patients with moderate illness, and green identifies patients who are allowed to go home after a medical examination.

**Figure 5.**
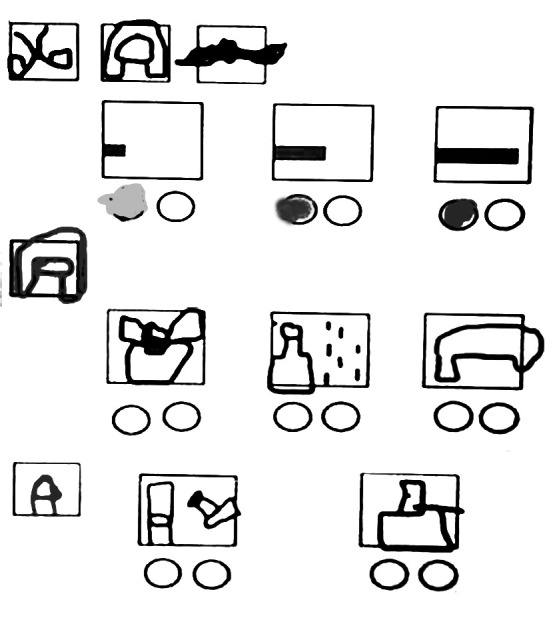
The modeling of *Beauty Salon*

*Figure 5* presents a play in which complex modeling of a beauty salon can be observed. The children prepared the symbols for attending to clients who came to get their hair cut, dyed, or styled; their nails polished; and so on. All the signs were created by the children together during discussion online; each child has drawn the symbols on the board at home and shown them to the other participants.

In this example, the upper three squares show the “services of hair cutting, hair-dressing, hair styling, and barber service.” The next line shows the “length of hair” that each “client” wishes to have: short, medium, or long. The line in the middle shows the possibilities of “hair styling” as “stylish combing, hair washing, and hair drying.” The bottom line shows the service only for nails: “cutting or painting.” The circles with the colors show the price for each service. This complex model was created during online discussion and play by children in front of the screen.

In these examples, we see how the online modality of playing with roles might be used as a methodology for preschool children. The children use the objects and toys in their own homes, but, with the guidance of the teacher, can explain, introduce, and show the other children how to create different symbolic means. One interesting aspect of the methodology is that the children not only see examples and symbols on the screen as if they were in a movie, but they take part in creating all the details. The children talk to each other throughout the preparation of the play and during the play-acting itself.

**Figure 6.**
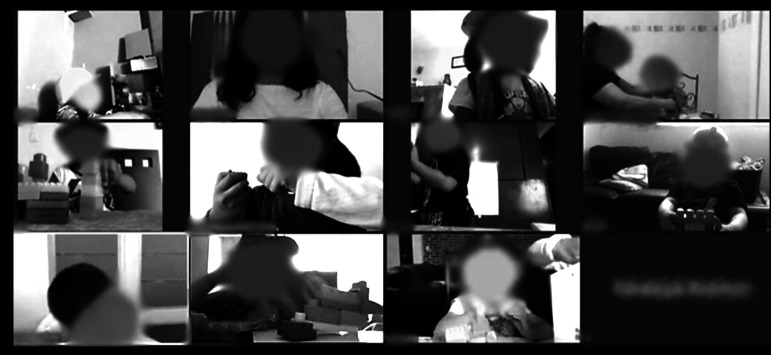
The play *Builders*

*Figure 6* presents another example of the process of producing a play with roles online.

The figure shows how the children represented the play’s roles by using concrete objects and toys and symbolic means for the roles of the builders, and blocks for the constructions of buildings. The children acted as “builders,” choosing the models and materials for design of the buildings and towers in an imaginary city.

## Discussion and Methodological Reflection

The question of the optimal ways and time for preparation to introduce mathematical knowledge in childhood remains unsolved in psychology. This article has compared the constructivist and cultural-historical approaches to this important problem. Some examples of concrete implementation of the cultural-historical methodology at preschool age were presented. Online fulfilment of the actions of the roles in the play allowed the children to create and to use the symbols in each play. Such experience is essential for future learning of mathematics in primary school (Komarova, 2022; Rosas, 2019, 2021; Salmina, 1988). The use of symbolic means was achieved using actions of codification and modeling of the situation in the play.

It is possible to observe that while putting on a play in the online modality, the process of introduction and use of symbols has some particular features. There is no place for substitution of objects, since the children are showing and using their toys and interesting objects according to the topic of the play and the actions of the characters. Codification and regulation are also modified by the concrete and mechanical manipulation of the participants, who have to take turns for oral expression and action on the screen. The turns are regulated by the naming of each participant, as the children, one by one, answer the questions of the educator and of each other, or while they show action with their toys and objects on the screen. Schematization of the actions is complicated online, because there is no ability to move about or to perceive the movements of the others on screen, but some of our concrete examples of schematization are shown in Figures 3–5. Modeling is acceptable for use online; however, the difficulty is that this level requires previous consistent work with the other levels. All these levels or types of symbolic actions should be understood by the children, so that they are able to propose different options, ideas, and solutions for each symbolic action.

The present study shows that the only real possibility for introducing symbolic functions is at the levels of codification and modeling of the situation in the play. The problem with the modeling level is that it is the most complex level of the development of symbolic function for preschool children. Not all children and not all grades of preschool education are prepared for this level of development of symbolic function. The level of modeling requires not only the representation or regulation of one’s behavior, such as following a rule in a table game, but also requires the children’s reflection about the elements of the material situation. This means that only in the third level of preschool education (when the children are between five and six years old) does the use of symbols become possible, as compared to the face-to-face modality, where the other levels of symbolic function have been included in our research starting with the second level of preschool education (the age between four and five years).

The use of symbolic means allowed the children not only to satisfy their curiosity and be positively engaged in the topic of the play, but also to develop symbolic functions as the basis for preparing for intellectual actions with numerical content in primary school. At the same time, it is necessary to be conscious that the effect of such development could never be compared with the same project carried out face-to-face.

There are some common and differential features between the constructivist and cultural-historical approaches to preschool learning of basic mathematical abilities.

The common feature of these two approaches is their rejection of explicit formal teaching of mathematics at preschool age. The constructivist approach chooses implicit inclusion of mathematical knowledge in actions of manipulation and exploration individually by each child. In this case, mathematical knowledge “is included” in the external materials and space of the classroom, such as magnitudes, quantities, measuring, seriation, and so on. All this content of basic mathematics is never presented directly to the child and never explained. That means that, during manipulation and exploration, the child learns nothing about magnitude, quantities, measuring, seriation, and correspondence. At the same time, there is no goal set for play activity, so that the child never knows how to play and what the purpose of the play is. The child is unconscious of all this implicit content of mathematical knowledge.

An interesting question remains: Is the teacher conscious of that content? How is it possible that the content, of which the child is unconscious, should pass into the consciousness of the child? If the whole process of developmental transition from unconscious to conscious knowledge is not clear at all, it becomes completely mysterious when teaching online. All the sensory conditions that are supposed to facilitate the discovery of mathematical knowledge by the child independently are simply absent in the online modality, as the child has no contact with external objects.

Another important feature of the constructivist position is that the children work in isolation, as each child completes his or her own independent manipulation. The goal of this manipulation is not clear from the beginning to the end of the operation. It looks as if constructivism claims that implicit intuitive knowledge is the unique way of learning at preschool and primary school. The essential question remains unsolved: How might that alleged intuitive knowledge be converted into clear and conscious knowledge in adolescence? How might free play and manipulation automatically prepare, without any goal or reflection, the older child’s mathematical abilities? A possible answer is that children either remain on the level of unclear intuition (the best variant) or totally lack any kind of mathematical knowledge (the worst option). The outcome is the poor level of success in learning mathematics in Mexico and other Latin American countries during international evaluations (OGDE, 2016).

The cultural-historical approach chooses another solution to the introduction of mathematical concepts. The process of teaching and learning is studied based on a unique psychological conception of development (Galperin, 2000; Podolskiy, 2009; Talyzina, 2018, 2019; Vygotsky, 1996). There is no place for implicit work with sensory isolated characteristics and operations. According to this conception, the preschool period of childhood should be dedicated to profound development of the symbolic function as the central element of preparation for learning mathematics in primary school (Salmina, 2013 a, b). According to Talyzina, playing activity should introduce and develop voluntary use of means and establishment of conscious goals in child’s activity (Talyzina, 2019). According to this author, the possibility for establishing of conscious goals is central for psychological preparation for school learning. So, the child’s inclusion in collective playing activity not only with distribution of roles, but also with distribution and creative of symbolic means is the best way for preparation of the child for learning at primary school.

The cultural-historical approach opens new possibilities for pedagogical work, including playing with roles online; it is one possible way for forming symbolic actions and providing preparation for introduction of mathematics in primary school (Sidneva et al., 2021; Solovieva & Quintanar, 2021). The accessible symbolic operations to be used in online sessions with groups of preschool children are operations of codification and modeling. The symbolic operation of codification is the most common operation in this modality; it is very attractive for children, and they may spend a long time designing different symbols for representation of rules, details, and content. The operation of modeling is rather complex and requires the use of external objects and of space. However, it is possible to introduce the children to the possibility of modeling an entire imaginary situation in a play featuring roles. As for the operations of substitution and schematization, they are rather complicated to be worked on in the online modality, as their proper inclusion requires movement and the use of different objects in real space during social interaction.

In our opinion, face-to-face sessions of play are ideal opportunities for the psychological development of preschool children. At the same time, online play sessions are useful for development of the symbolic function. To enrich the constructivist approach applied in Mexico, other possible symbolic operations should be considered for preschool age children, to provide better levels of preparation for the introduction of mathematics in primary school according to the cultural-historical approach and activity theory applied to the teaching and learning process.

The work during the pandemic allowed the authors to systematize the points of coincidence and fundamental difference between constructivism and the cultural-historical approach. *Table 2* presents this systematized comparison between the two approaches to work on mathematics with children of preschool age.

**Table 2:** Comparison of constructivist and cultural-historical approach for preschool age

Content of comparison	Constructivist approach	Cultural-historical approach
Play activity	Free play	Organized thematic play with roles and rules
Plan of actions	Sensory empirical operations of manipulation or exploration with no distinction between concrete objects and symbols	Actions with material objects and toys, actions of materialized representation, creation and use of perceptive symbols, creation of schemes and plans
The role of an adult	Facilitation, observation, communication	Orientation for all elements of content of the play activity
Communication	Positive inclusive communication with no specific goals in free play and sensory exploration	Positive inclusive communication directed to fulfilment of the goals of play activity
Work with mathematical abilities	Implicit inclusion of empirical ideas of length, area, volume, weight, quantity, measuring, digits	Reflection on the necessity of use and creation of symbolic means as the content for representation, schematization, codification, and modeling in play activity; reflection about diversity of external, perceptive, and verbal symbols
Psychological results	High level of sensory manipulation and exploration without specific goal, no differentiation between plan of representation and means of representation	High level of development of symbolic function, reflection, voluntary activity, and imagination

The most notable differences between the constructivist and cultural-historical approaches consist in the use of play activity and the inclusion of symbolic means in this activity (Bredikyte, 2012; Hakkarainen & Bredikyte, 2020). The work with symbolic functions is practically absent in the constructivist approach because the use of symbolic means is implicit. In this approach, the children never know reflectively whether they are using concrete sensory objects or symbols during manipulation.

On the contrary, according to the cultural-historical approach, the children who take part in the plays with roles not only understand the representative role of the symbols, but also may create them and use them collectively for a common purpose. Different methods for psychological development in preschool age provide different levels of readiness for school (Nisskaya, 2018; Sidneva, 2020; Nasledov et al., 2020). The conditions of educational sessions online during the pandemic allowed us to work within the cultural-historical conception of development and offer play featuring role activity with inclusion of symbolic means. The constructivist approach in Mexico has no specific proposals for development under the conditions of pandemic, only offering the strategy of sending tasks, orally or as video, to the parents by WhatsApp. Very few preschool institutions in Mexico provided real simultaneous online sessions under the conditions of the pandemic.

Our future research will be directed to creation and testing of original methods for introduction of intellectual action and basic mathematical knowledge in primary school according to the cultural-historical approach and activity theory.

## Conclusions

The constructivist and the cultural-historical approaches are two different alternatives to the traditional, repetitive way of teaching mathematics at preschool age. Both methods provide an atmosphere of collaboration and positive affective communication for the children. The difference resides in the apprehension of the role and the content of the symbolic function in preparation for formal studies of mathematics. The constructivist position is based on free play and sensory exploration at preschool age and implicit work, with no reflection about the difference between practical and intellectual actions.

The cultural-historical approach proposes the introduction of organized thematic play with roles in preschool, which offers broad possibilities for symbolic development as a necessary platform for formal learning of mathematics in primary school. According to the cultural-historical conception, online sessions for preschool education might be organized as collective activities guided by the teacher. Such simultaneous online play sessions should include participation by the teacher and the children with the broad use of collective dialogue, material objects, drawings, and symbolic means, which require the proper introduction of gradual levels of symbolic operations: substitution, codification, schematization, and modeling.

## Limitations

The main limitations of our study are related to the impossibility of face-to-face work during the international pandemic and to the small groups of participants, which do not allow us to reliably declare statistical results.
